# Metastatic Sclerosing Epithelioid Fibrosarcoma at Diagnosis: A Case Report

**DOI:** 10.7759/cureus.75544

**Published:** 2024-12-11

**Authors:** Isabel R Miguel, Mavilde Arantes, Rafael Matias, Ana M Ferreira, Mariana Afonso

**Affiliations:** 1 Radiation Oncology, Instituto Português de Oncologia do Porto Francisco Gentil, Porto, PRT; 2 Neuroradiology, Instituto Português de Oncologia do Porto Francisco Gentil, Porto, PRT; 3 Oncology, Instituto Português de Oncologia do Porto Francisco Gentil, Porto, PRT; 4 Pathological Anatomy, Instituto Português de Oncologia do Porto Francisco Gentil, Porto, PRT

**Keywords:** metastatic sarcoma, oncology, palliative care, radiation oncology, sclerosing epithelioid fibrosarcoma

## Abstract

Sclerosing epithelioid fibrosarcoma (SEF) is a rare and aggressive neoplasm composed of epithelioid cells arranged in strands and nests embedded in a highly sclerotic collagenous stroma. We report a case of a 36-year-old man who started with lumbar pain, with extension to both legs, night sweats, and weight loss. He underwent magnetic resonance imaging (MRI) of the lumbar spine; computed tomography (CT) scan of the chest, abdomen, and pelvis; and [18F]-fluorodeoxyglucose positron emission tomography/computed tomography (18F-FDG PET/CT) scan. The CT scan revealed a 13 cm thoracic mass, the MRI presented with diffuse neoplastic invasion of the vertebrae, and the PET showed hepatic, bone, and possibly pulmonary metastases. The histological diagnosis was compatible with SEF. The disease progressed very quickly, namely, with an episode of spinal cord compression, which made the patient paraplegic. He underwent surgery and, subsequently, radiotherapy (RT). Due to the clinical and analytical evolution, it was not possible to initiate systemic treatment and the patient ultimately passed away. In conclusion, SEF is an aggressive type of sarcoma that affects middle-aged patients, with high rates of distant metastases and mortality. The usual treatment is surgery followed by either radiotherapy or chemotherapy. However, further clinical trials are needed to find more systemic target therapies.

## Introduction

In 1995, Meis-Kindbloom recognized the term “sclerosing epithelioid fibrosarcoma” (SEF) as a distinctive form of low-grade soft tissue sarcoma [1]. Currently, the World Health Organization (WHO) recognizes it as a distinct entity [2]. The most frequent sites of involvement are deep soft tissues of the lower extremities and limb girdle [3,4].

SEF is a low-grade neoplasm, characterized by the proliferation of round to oval epithelioid cells, normally with uniform nuclei, small to moderate in size, with a clear cytoplasm. The cells are organized in cords, trabeculae, and nests and are immersed in a stroma, which is usually sclerotic but can have areas of myxoid changes, the presence of hyaline cartilage, calcifications, and metaplastic bone formation. In some cases, the tumor can contain areas of spindle cells, resembling “fibrosarcoma-like areas” [1,3,4].

Immunohistochemically, MUC4 (mucin 4) is diffusely and strongly positive, representing a sensitive and specific marker for SEF. Other immunohistochemical markers that can be positive are CD99, BCL-2, EMA, and vimentin [4,5].

Concerning molecular changes, pure SEF has recurrent EWSR1 gene rearrangements, predominantly EWSR1::CREB3L1 fusion and, less frequently, EWSR1::CREB3L2. Considering morphological and genetic data, it is assumed that there is a link between SEF and low-grade fibromyxoide sarcoma (LGFMS), and cases of hybrid SEF/LGFMS are more likely to carry FUS-CREB3L2 [3-5].

This report is about a case of an adult man diagnosed with SEF in an advanced stage.

This case was previously presented as a meeting abstract for the 2024 Oncology Spring Meeting, held in Portugal from April 10 to 13, 2024.

## Case presentation

A 36-year-old male patient, a high-ranking volleyball player, presented with lumbar pain radiating to both legs, progressively worsening over several months, which he initially attributed to intense physical activity. The patient also reported night sweats and unquantified weigh loss.

His past medical history included only a left shoulder orthopedic surgery one year earlier, following a bike accident. He had a son with multiple osteochondrosis.

**Figure 1:**
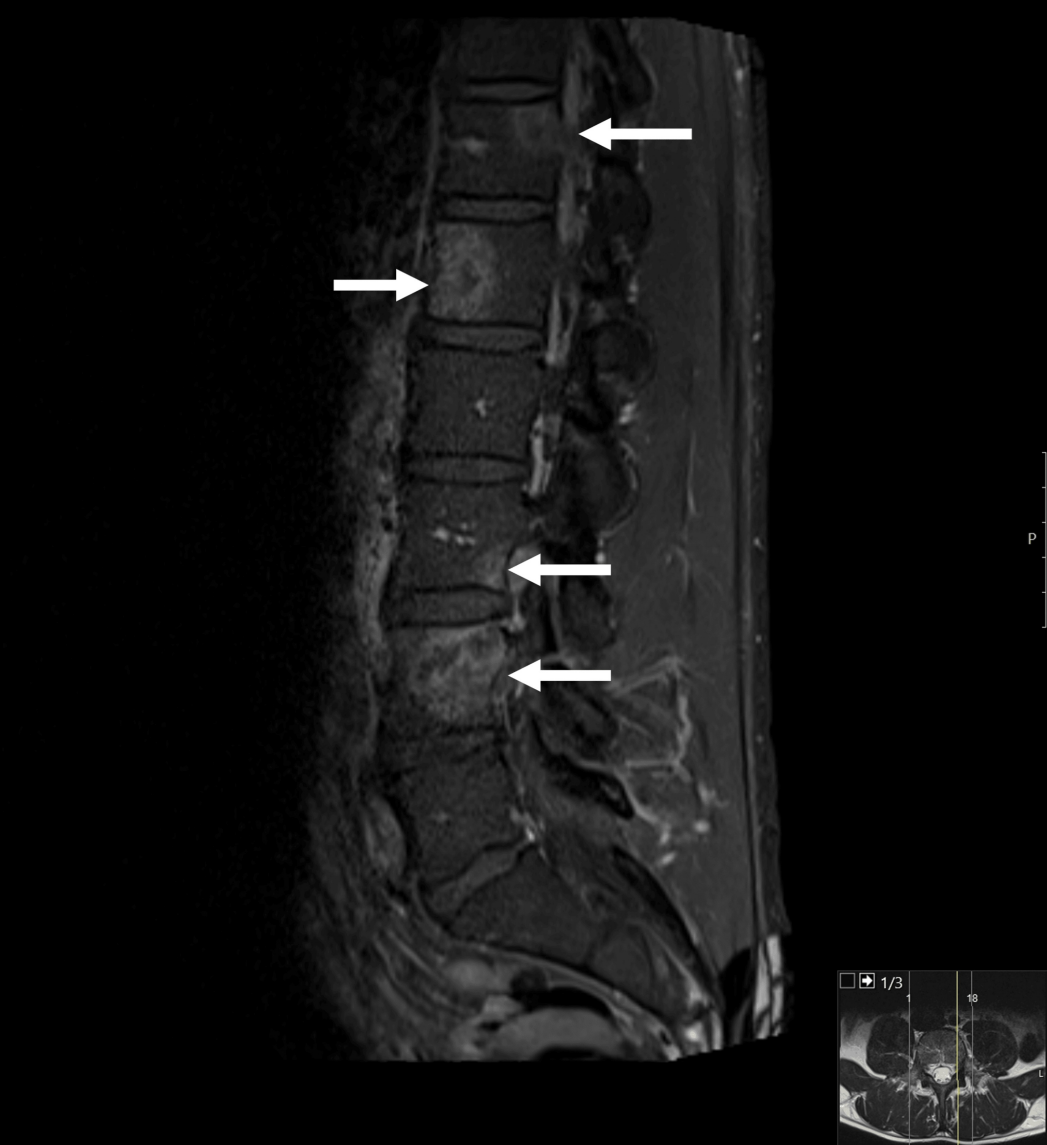
Magnetic resonance imaging T1-weighted imaging (MRI T1W1) sequence: sagittal plane Neoplastic invasion of the vertebrae of D12, L1, L3, and L4 (arrows)

**Figure 2:**
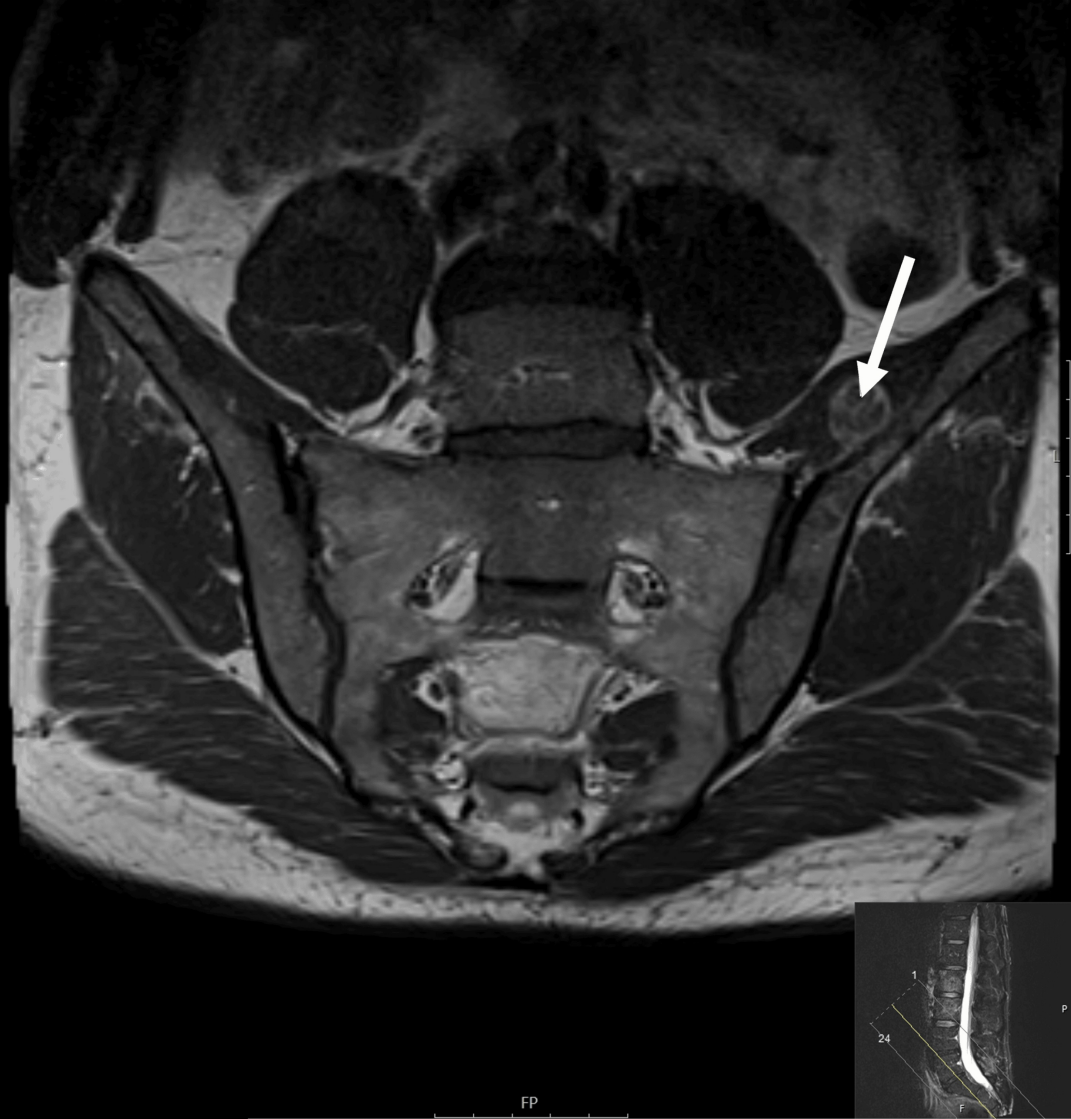
Magnetic resonance imaging T1-weighted imaging (MRI T1W1) sequence: axial plane Neoplastic invasion of the sacrum and of the iliac bone, with an extension to the iliac muscle (arrow)

A magnetic resonance imaging (MRI) of the lumbar spine (October 2022) showed neoplastic invasion of the vertebrae of D12, L1, L3, and L4, mainly centered on the trabecular bone (Figure 1). There was also involvement of the sacrum (bilaterally) and the iliac bone (mainly of the left), the latter with an extension to the iliac muscle (Figure 2). These lesions were interpreted as metastases. 

**Figure 3:**
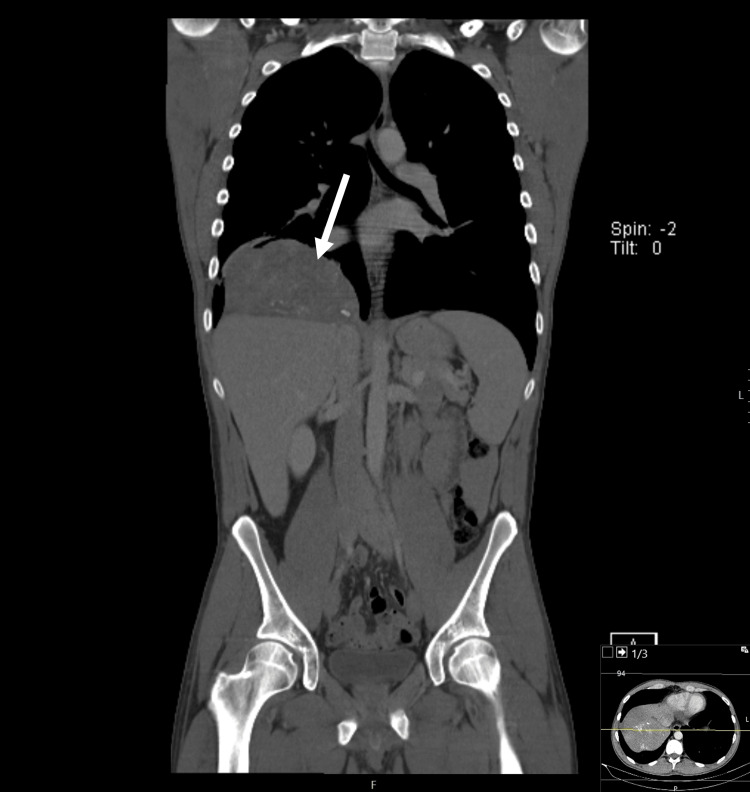
Chest, abdomen, and pelvic computed tomography (CT): coronal plane Hypodense mass, adjacent to the right part of the diaphragm and with extension into the thoracic cavity

**Figure 4:**
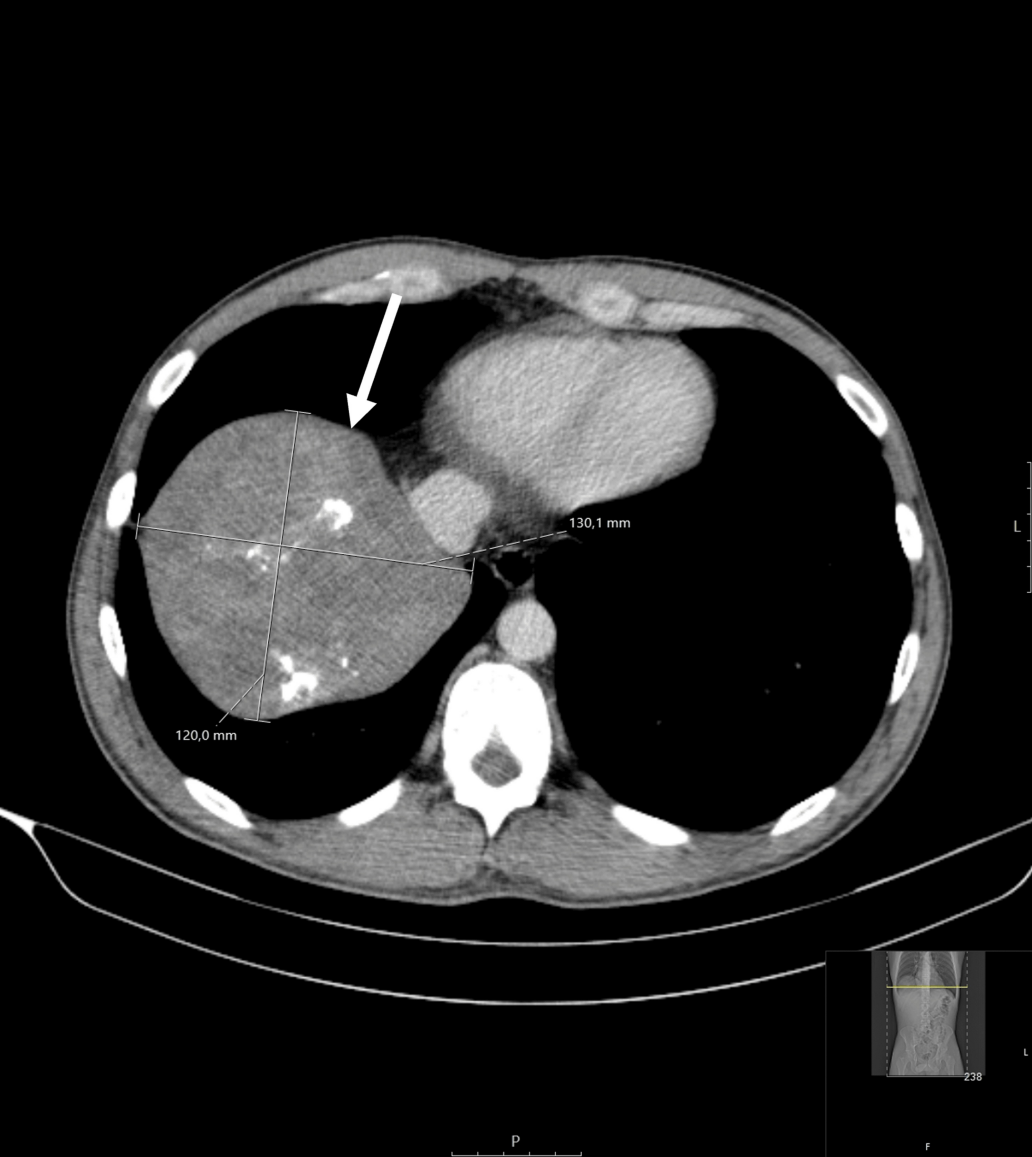
Chest, abdomen, and pelvic computed tomography (CT): axial plane Hypodense mass extending into the thoracic cavity, with areas of calcification, measuring 12 x 13 cm

A chest, abdomen, and pelvic CT (October 2022) revealed a 12 x 13 cm hypodense mass adjacent to the right diaphragm, extending into the thoracic cavity. The mass had the density of soft tissue, with some contrast enhancement and areas of calcification (Figures 3, 4). In addition, there was a micronodule in the inferior left pulmonary lobe and two hypodense nodular hepatic lesions (in segments V and VI). 

In November 2022, the patient underwent a biopsy of the thoracic mass outside our institution, which raised suspicion of a mesenchymal tumor.

**Figure 5:**
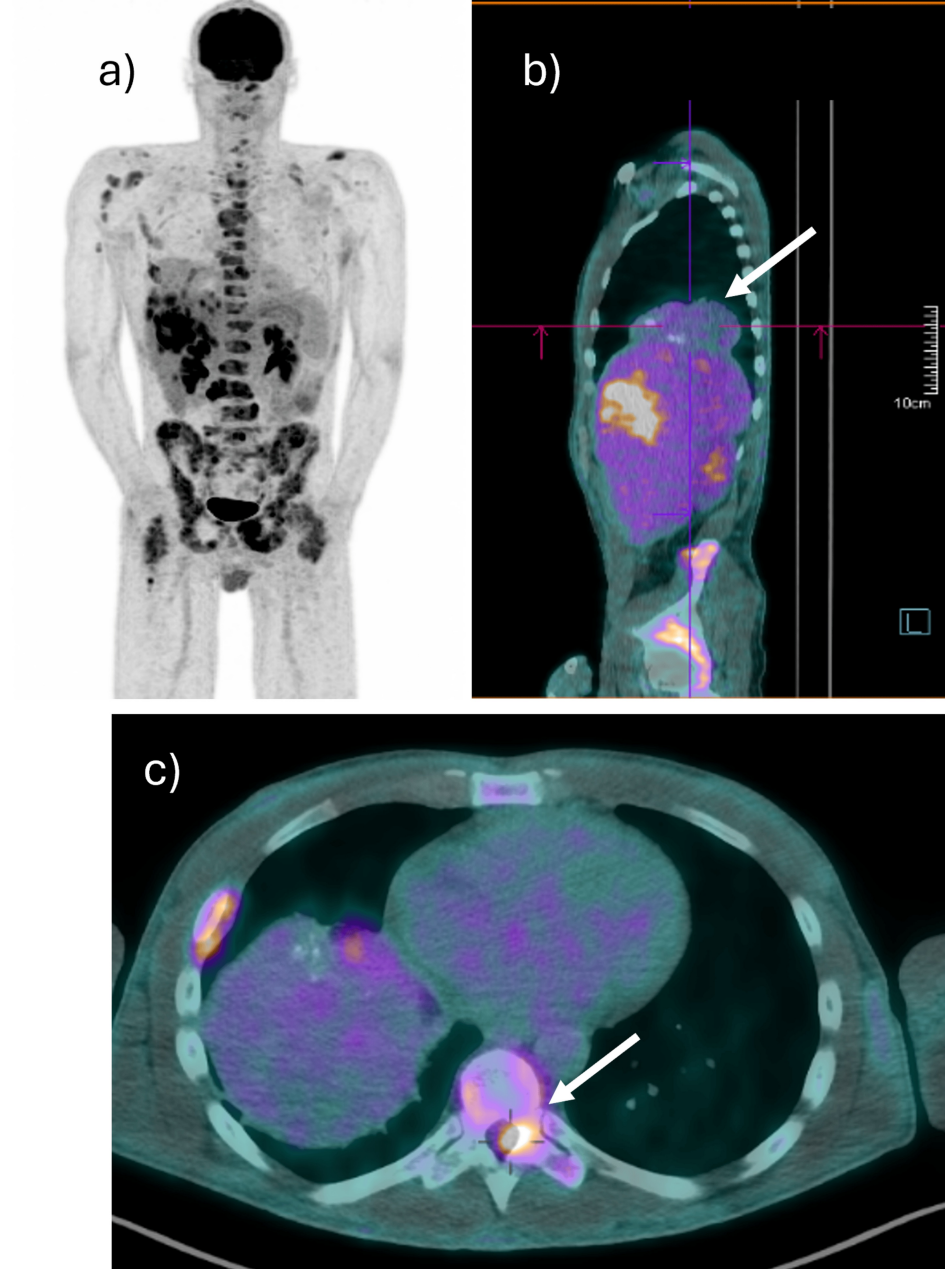
[18F]-fluorodeoxyglucose positron emission tomography/computed tomography (18F-FDG PET/CT) a) Disseminated neoplasic involvement; b) thoracic mass (arrow); c) spinal canal lesion at the level of D9 (arrow)

In December 2022, an [18F]-fluorodeoxyglucose positron emission tomography/computed tomography (18F-FDG PET/CT) scan was performed, which identified a bulky thoracic mass with moderate and heterogeneous uptake. It also revealed disseminated hepatic metastases and bone involvement (spine, pelvis, sternum, clavicles, shoulder blades, ribs, and proximal portion of the humerus and femur). There was also infiltration of the soft tissues (right psoas muscle and the internal and external obturated muscles), and a probable lesion with extension into the spinal canal at the level of D9. In addition, several pulmonary micronodules were noted, likely metastatic (Figure 5).

In December 2022, the patient had an episode of acute spinal cord compression, which presented with acute paraplegia and urinary retention.

The MRI confirmed the presence of an extramedullary spinal canal expansive lesion, at the level of D8-D9, hyperintense in T2WI acquisition, compressing the spinal cord. The lesion had an extension to the left intervertebral foramen (indenting the root structures) and to the paraspinal soft tissue. There was another lesion, at the level of L3-L4, also extradural (slightly compressing the thecal sac) and with extension to the intervertebral foramina (indenting the root structures at the level of L3).

A surgery of spinal decompression was performed, with laminectomy of D9 and excision of the spinal canal lesion of D8-D9. The patient never recovered from the deficits.

He was admitted to our institution after attending the emergency department (one month after the surgery, in January 2023), with non-controlled right shoulder pain and decreased functional capacity of the upper right limb. At this point, he was paraplegic and using a vesical catheter chronically. At the physical examination, he had a 2 cm hard-elastic left supraclavicular adenopathy.

During hospitalization, a cranioencephalic MRI showed neoplastic infiltration of the skull base bones (particularly of the left occipital condyle), with a cervical soft tissue component and with extension to the hypoglossal nerve. A complete MRI of the spine showed once again diffuse bone metastases, with soft tissue involvement of multiple levels, namely, D2, D8, D9, D11, L3, and L4.

**Figure 6:**
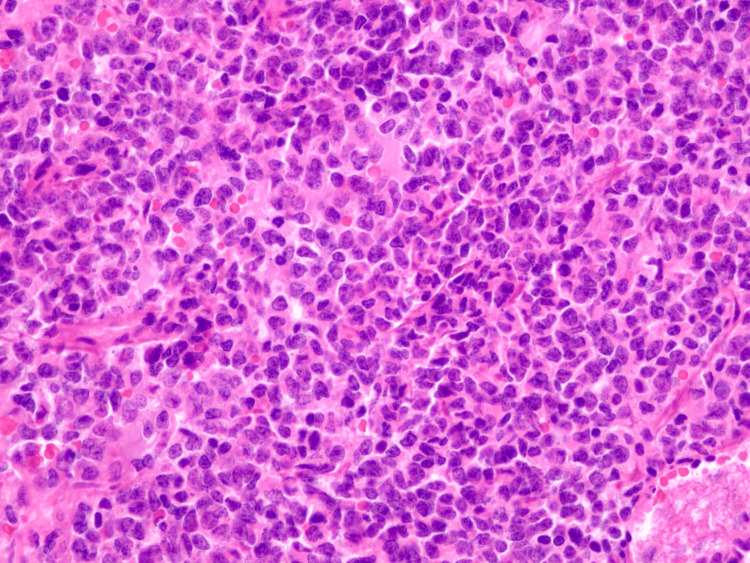
Histological evaluation: hematoxylin and eosin (H&E) staining Malignant neoplasm composed of epithelioid cells, disposed in sheaths and trabecula in a dense and sclerohialinized stroma

**Figure 7:**
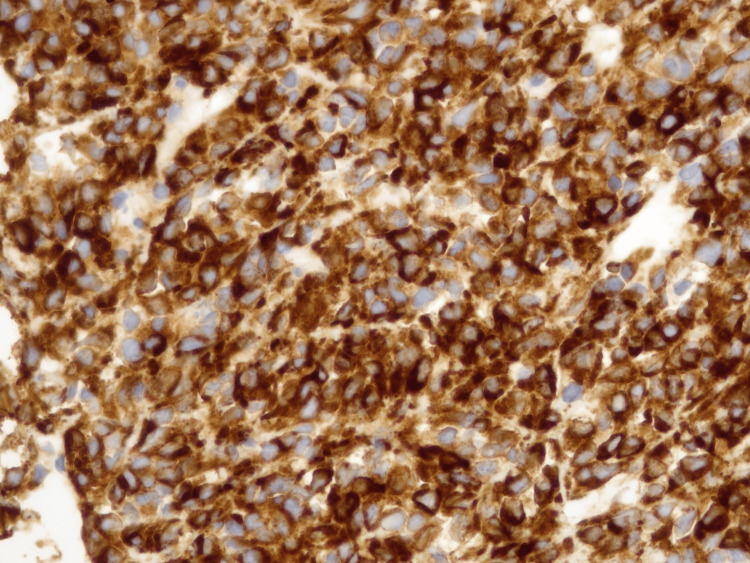
Immunohistochemical study Strong and diffuse positivity for MUC4

The histological reevaluation of the spinal decompression product was performed at our institution, and a diagnosis of SEF was rendered (Figure 6). The immunohistochemical study showed diffuse positivity for MUC4 (Figure 7) and the genetic analysis with the next-generation study (NGS) using “TruSight RNA Fusion Panel” revealed fusion EWSR::CREB3L2. 

The biopsy of the cervical adenopathy and the hepatic lesion were compatible with the metastatic involvement of SEF.

The histology of the diaphragmatic/thoracic mass biopsy, also reviewed in our institution, although conditioned by the scarcity of material, revealed a neoplasm with morphologic and immunohistochemical characteristics compatible with the involvement of the SEF.

**Figure 8:**
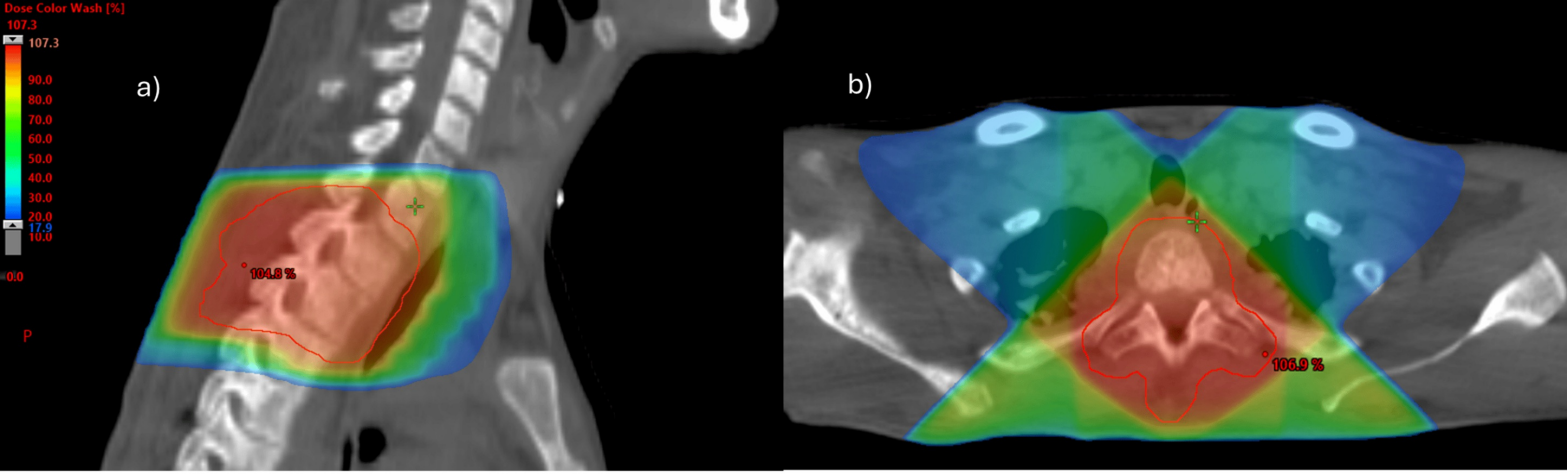
Radiotherapy treatment plan – spine lesions of D2-D3 Colorwash distribution of dose: sagittal (a) and axial (b) planes. The red line corresponds to the planning target volume (PTV).

**Figure 9:**
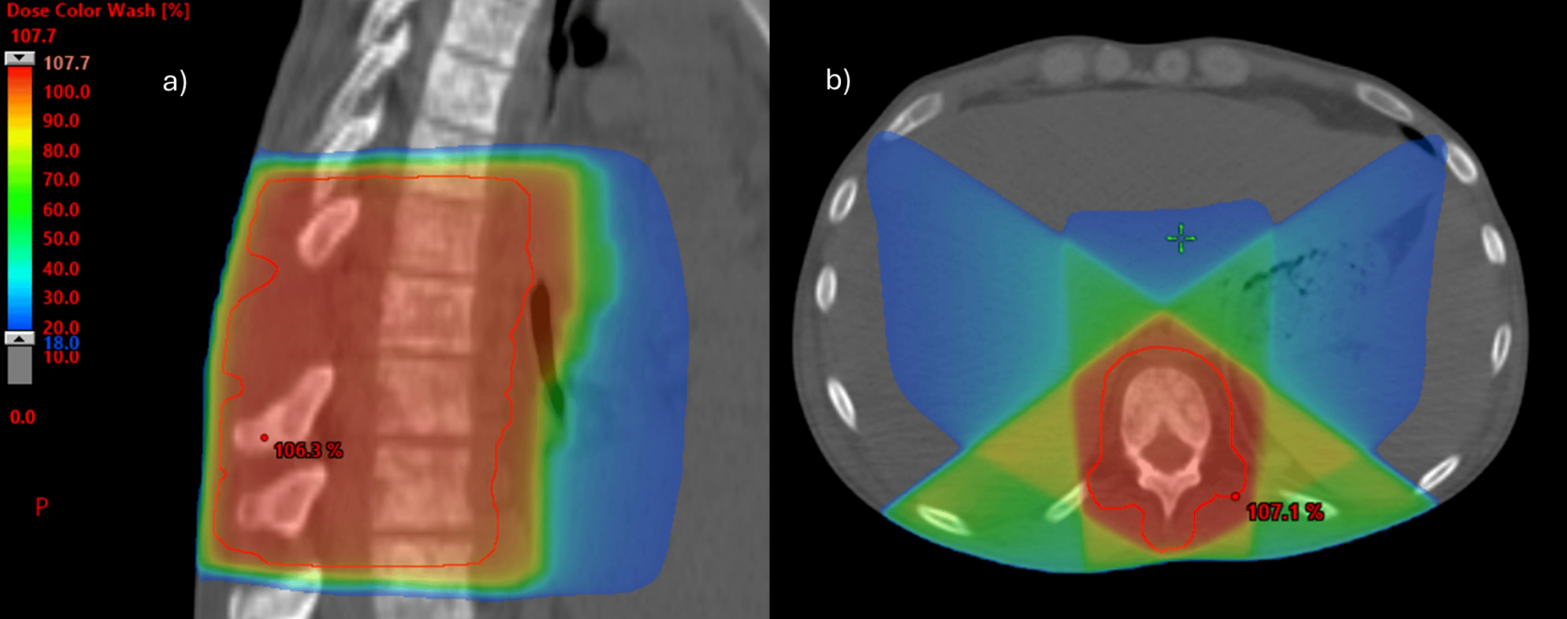
Radiotherapy treatment plan – spine lesions of D8-D11 Colorwash distribution of dose: sagittal (a) and axial (b) planes. The red line corresponds to the planning target volume (PTV).

During hospitalization, he was submitted to consolidative/antalgic radiotherapy (from February 1, 2023, to February 16, 2023) in a total dose of 36Gy in 12 fractions, with the three-dimensional conformal radiation therapy (3DCRT) technique, to the spine lesions at the levels of D2-3 (Figure 8) and D8-D11 (Figure 9). The treatment occurred without any complications and with good pain tolerance. 

This palliative radiotherapy fractionation scheme was chosen because the tumor is a sarcoma. Due to being relatively radio-resistant tumors, higher effective biological doses than those typically used for most solid tumors are usually required to achieve symptomatic control.

After the histological diagnosis, it was decided at the tumor board (February 2023) that the patient would be submitted to systemic therapy and included in the institution program for precision oncology (for a possible target therapy).

During hospitalization, the patient developed pancytopenia: first anemia (normocytic and hypoproliferative, due to chronic disease), then leukopenia (with a neutropenia graded 4/4), and, lastly, thrombocytopenia. 

Besides this, during hospitalization, he had a urinary tract infection and several episodes of fever. The patient did not manage to start any systemic therapy, due to this analytical evolution.

The patient ultimately passed away at the end of February 2023.

## Discussion

SEF is a rare distinct variant of fibrosarcoma that affects most commonly middle-aged individuals. It has an aggressive evolution, despite the relatively bland morphology. SEF tends to have an aggressive clinical course, with rates of recurrence of 27%, distant metastases of 80%, and mortality of 47% [4,5].

The primary location is normally the soft tissues of the upper or lower extremities, followed by the trunk, head, and neck [4]. Some studies show other possible locations, such as the kidneys, lungs, pelvis, uterus, pancreas, and stomach [5].

The bone is a rarer primary location for SEF, and due to its rarity, the differential diagnosis appears to be more challenging. It is frequently misdiagnosed due to its resemblance to other common bone sarcomas, particularly osteosarcoma and Ewing sarcoma [6,7]. There are few published cases of primary bone SEF. However, there is no consensus on the most common primary bone location. Some articles point to long bones [6,7], some to the spine [8], and others to flat/irregular bones [9].

The metastases of SEF most commonly affect the lungs, followed by the bone, lymph nodes, pleura, and brain [4].

We present a case of an adult man who was diagnosed with advanced SEF. At the time of diagnosis, besides a bulky thoracic mass, he already had diffuse involvement of the bone, with soft tissue components in some locations. He also had hepatic, and probably pulmonary, metastases. During the follow-up, the sarcoma progressed, namely, to the skull base bones and lymph nodes.

The diagnosis of SEF was based on the histologic, immunohistochemical, and genetic results, namely, the diffuse positivity for MUC4 and the genetic fusion EWSR::CREB3L2. As the neoplasia was diagnosed in such an advanced stage, it was not possible to perform surgery as a curative treatment. He underwent surgery in an emergency context, due to spinal cord compression syndrome, and after that, he was submitted to consolidative/antalgic radiotherapy. Although it was initially planned to do systemic therapy, the clinical and analytical evolution was an obstacle.

Histologically, it was not possible to say with certainty whether the primary lesion was either from the bone or from the soft tissue. However, considering the radiological characteristics of the lesions, the bone involvement was more likely to be secondary and the thoracic mass more likely to represent the primary tumor.

Reviewing the literature, the thoracic cavity is a rare location for the primary involvement of SEF, which makes this case quite unique. To our knowledge, there are five published cases of a primary SEF of the chest wall [10-13], one case of primary SEF of the pleura [14], and one of primary SEF of the lung [15]. In the case we present, although the mass did not have a completely defined anatomical limit, it was related to the right lower chest wall.

The treatment of single-sited SEF normally includes surgery followed by radiotherapy and/or chemotherapy [5]. However, the studies show limited response to conventional chemotherapy agents, namely, in the advanced stage [4,16,17]. Some studies already suggest potential therapeutic targets, such as CD24 and the DMD gene [18], which justifies the request for genetic analysis in the diagnostic approach. Further investigation is needed to understand the underlying disease biology and identify novel therapeutic options, to improve the prognosis of this entity.

## Conclusions

SEF is an aggressive type of sarcoma with high rates of metastases and mortality. The diagnosis is difficult because it can be expressed by different patterns with varying cell morphology. The rarity of the disease and the difficult positive histological diagnosis are limitations for a better understanding of the disease. The treatment is also a challenge, with the need for future investigation, particularly at the level of targeted systemic therapies.
